# Pandemic Response Box^®^ library as a source of antifungal drugs against *Scedosporium* and *Lomentospora* species

**DOI:** 10.1371/journal.pone.0280964

**Published:** 2023-02-03

**Authors:** Rodrigo Rollin-Pinheiro, Mariana Ingrid Dutra da Silva Xisto, Yuri de Castro-Almeida, Victor Pereira Rochetti, Luana Pereira Borba-Santos, Yasmin da Silva Fontes, Antonio Ferreira-Pereira, Sonia Rozental, Eliana Barreto-Bergter

**Affiliations:** 1 Laboratório de Química Biológica de Microrganismos, Departamento de Microbiologia Geral, Instituto de Microbiologia Paulo de Góes, Universidade Federal do Rio de Janeiro, Rio de Janeiro, Brazil; 2 Programa de Biologia Celular e Parasitologia, Instituto de Biofísica Carlos Chagas Filho, Universidade Federal do Rio de Janeiro, Rio de Janeiro, Brazil; 3 Laboratório de Bioquímica Microbiana, Departamento de Microbiologia Geral, Instituto de Microbiologia Paulo de Góes, Universidade Federal do Rio de Janeiro, Rio de Janeiro, Brazil; University of Sydney, AUSTRALIA

## Abstract

*Scedosporium* and *Lomentospora* species are opportunistic filamentous fungi that cause localized and disseminated infections in immunocompetent and immunocompromised patients. These species are considered resistant fungi due to their low susceptibility to most current antifungal agents used in healthcare settings. The search for new compounds that could work as promising candidate antifungal drugs is an increasing field of interest. In this context, in the present study we screened the Pandemic Response Box^®^ library (Medicines for Malaria Venture [MMV], Switzerland) to identify compounds with antifungal activity against *Scedosporium* and *Lomentospora* species. An initial screening of the drugs from this collection at 5 μM was performed using a clinical *Scedosporium aurantiacum* isolate according to the EUCAST protocol. Compounds with activity against this fungus were also tested against four other species (*S*. *boydii*¸ *S*. *dehoogii*, *S*. *apiospermum* and *L*. *prolificans*) at concentrations ranging from 0.078 to 10 μM. Seven compounds inhibited more than 80% of *S*. *aurantiacum* growth, three of them (alexidine, amorolfine and olorofim) were selected due to their differences in mechanism of action, especially when compared to drugs from the azole class. These compounds were more active against biofilm formation than against preformed biofilm in *Scedosporium* and *Lomentospora* species, except alexidine, which was able to decrease preformed biofilm about 50%. Analysis of the potential synergism of these compounds with voriconazole and caspofungin was performed by the checkerboard method for *S*. *aurantiacum*. The analysis by Bliss methodology revealed synergistic effects among selected drugs with caspofungin. When these drugs were combined with voriconazole, only alexidine and amorolfine showed a synergistic effect, whereas olorofim showed an antagonistic effect. Scanning electron microscopy revealed that alexidine induces morphology alterations in *S*. *aurantiacum* biofilm grown on a catheter surface. Reactive oxygen species production, mitochondrial activity and surface components were analyzed by fluorescent probes when *S*. *aurantiacum* was treated with selected drugs and revealed that some cell parameters are altered by these compounds. In conclusion, alexidine, amorolfine and olorofim were identified as promising compounds to be studied against scedosporiosis and lomentosporiosis.

## Introduction

*Scedosporium* and *Lomentospora* species comprise filamentous fungi responsible for clinical manifestations ranging from superficial to invasive infections and are considered emergent pathogens due to the increase of their incidences over the last decades, especially in Europe, Oceania, America and Asia [1, 2]. Superficial infection cases are related to localized infections found in immunocompetent patients and consist of the formation of mycetoma, which is a cutaneous and subcutaneous malignancy that can result in the amputation of the affected member in cases of inappropriate or absent treatment [1]. In the context of invasive infections, risk factors usually associated with scedosporiosis and lomentosporiosis are organ transplantation, immunocompromising diseases including HIV/AIDS and near-drowning events [3]. In addition, both fungi are the second most frequent cause of pulmonary fungal infections in cystic fibrosis (CF) patients [4].

The treatment of fungal infections is a concerning challenge because only few antifungal drugs are available in clinical settings [5, 6]. Moreover, these drugs display significant levels of toxicity and side effects, which limit their use, especially in patients presenting underlying diseases. *Scedosporium* and *Lomentospora* species are considered resistant fungi due to their low susceptibility to most current antifungal agents used in healthcare settings, such as fluconazole and itraconazole [1, 7–11]. Amphotericin B is discouraged due to the high resistance of these pathogens [12]. Echinocandins, such as anidulafungin and caspofungin, are only recommended as salvage treatment, because both fungi are only weakly susceptible [7, 10, 13]. Voriconazole is suggested by the European Confederation of Medical Mycology (ECMM) as the first line of treatment of scedosporiosis and lomentosporiosis, since it presents the best susceptibility profile compared to other antifungal drugs [7].

Pathogenic fungi display a variety of resistance mechanisms to antifungal drugs, but little information is available for *Scedosporium* and *Lomentospora* species. Both fungi form biofilm structures, which are more resistant to antifungals when compared to planktonic cells [14]. Resistance of *Scedosporium* biofilms to some azoles, such as fluconazole, itraconazole and voriconazole, are more than 10-fold higher when compared to planktonic cells [14], suggesting that it could be an essential mechanism to protect these fungi against antifungal agents. Therefore, alternative treatments to improve the clinical management of scedosporiosis and lomentosporiosis are urgently needed.

The search for new compounds that are promising candidates as antifungal drugs is of increasing interest. A useful approach is the screening of libraries containing a variety of molecules, because this strategy optimizes the evaluation of a large number of compounds. One source for these libraries is the Medicines for Malaria Venture (MMV) organization, which provided two sets of compounds called Pathogen Box^®^ and Pandemic Response Box^®^, containing 400 compounds each [15]. The Pathogen Box^®^ has already been tested against some relevant pathogenic fungi, such as *Cryptococcus*, *Candida*, *Sporothrix* species, and chromoblastomycosis agents [16–20]. The Pandemic Response Box^®^ has also been tested against *Cryptococcus* species, *Candida auris* and some common eumycetoma causative agents [21, 22]. More recently, our group screened the library for *Scedosporium aurantiacum*, and identified two known compounds, iodoquinol and auranofin, as well as two non-commercial molecules which were also active against other *Scedosporium* and *Lomentospora* species [23]. These data show that the screening of libraries is a useful and promising approach to find alternatives for the treatment of infections caused by *Scedosporium* and *Lomentospora* species.

Considering the relevance of both species as emerging fungal pathogens, and the results reported in previous studies with the Pathogen Box^®^, the present study aimed to screen a total of 400 compounds from the Pandemic Response Box^®^, the second library from MMV, in order to identify additional candidates that present anti-*Scedosporium* and anti-*Lomentospora* activity.

## Materials and methods

### Strains and growth conditions

The following strains were kindly provided by Sybren De Hoog, from the Westerdijk Fungal Biodiversity Institute, Utrecht, the Netherlands: *Scedosporium aurantiacum* CBS 136046, *Scedosporium boydii* CBS 120157, *Scedosporium apiospermum* CBS 117407, and *Scedosporium dehoogii* CBS 117406. *Lomentospora prolificans* FMR 3569 was kindly provided by Dr J. Guarro, Unitat de Microbiologia, Facultat de Medicina e Institut d`Estudis Avançats, Réus, Spain. Fungal stocks were kept in modified Sabouraud medium (0.5% yeast extract, 1% peptone, and 2% glucose monohydrate). To obtain conidia for the experiments, cells were grown on modified Sabouraud agar plates for seven days at room temperature. Cell growth on the surface of the medium was scraped and suspended in sterile phosphate-buffered saline (PBS, pH 7.2). The suspension was filtered, centrifuged and cells were counted in Neubauer’s chamber, to be used in the experiments.

#### Compounds

The Pandemic Response Box^®^ library was provided by the Medicines for Malaria Venture organization and is composed of 400 compounds at 10 mM in dimethyl sulfoxide (DMSO). A stock solution of each compound was kept at 1 mM in DMSO and stored at −20°C. Voriconazole was used as a standard antifungal drug and was obtained from Sigma-Aldrich (Sigma Chemical Co., St. Louis, MO, USA).

#### Screening of the Pandemic Response Box library

*Scedosporium aurantiacum* CBS 136046 was used as reference strain to screen the Pandemic Response Box library due to its relevance as virulent and resistant isolate from the *Scedosporium* and *Lomentospora* species.

Screening was performed at 5 μM of each compound diluted in RPMI 1640 medium (Sigma Chemical Co., St. Louis, MO, USA) supplemented with 2% glucose and buffered with 3-(N-morpholino) propanesulfonic acid (MOPS) (0.165 mol/L, pH 7.2, from here on referred to as ‘supplemented RPMI’) in 96-well microtiter plates. Voriconazole (5 μM) and supplemented RPMI with DMSO 0.5% were used as controls. Conidia (2 x 10^5^/mL) were added and incubated for 72 h at 37°C in a 5% CO_2_ atmosphere. Fungal growth was quantified by optical density readings using a spectrophotometer (Bio-Rad, Hercules, CA, USA) at 600 nm. An inhibition of at least 80% was defined as a cut-off to select the promising drugs with antifungal activity against *Scedosporium* and *Lomentospora* species.

### Antifungal susceptibility testing

The susceptibility of *Scedosporium* and *Lomentospora* species to alexidine, amorolfine and olorofim was determined using the broth microdilution method, according to EUCAST protocols, with modifications [24]. Voriconazole was also used as a reference antifungal because it is the drug of choice for the treatment of scedosporiosis. Briefly, a serial dilution (10–0.078 μM) of each compound was prepared in supplemented RPMI 1640 medium in 96-well microplates. A standardized suspension of conidia (2 x 10^5^/mL) was added to each well and incubated for 72 h at 37°C, in a 5% CO_2_ atmosphere. Fungal growth was analyzed by spectrophotometry readings (Bio-Rad, Hercules, CA, USA) at 600 nm and cell viability was assessed using the XTT-reduction assay [25]. The minimum inhibitory concentration (MIC) of each compound was defined as the lowest concentration that inhibits 80% of fungal growth.

### Biofilm formation and preformed biofilm assay

Biofilm formation was analyzed according to Rollin-Pinheiro and colleagues [14]. Firstly, an adhesion step was performed by adding 200 μL from a standardized suspension of *Scedosporium* and *Lomentospora* conidia (1 x 10^7^/mL) to each well of a polystyrene microplate and incubated for 1.5 h at 37°C. Then, the supernatant containing non-adherent cells was removed and RPMI 1640 medium supplemented with MOPS, 2% glucose, and 20% fetal bovine serum (FBS, Gibco, Waltham, MA, USA) was added in the absence (positive control) or presence of selected compounds (8 - ¼ × MIC). Adherent cells were then incubated for 24 h at 37°C.

For the preformed biofilm assay, cells were cultured as described above in the absence of the compounds. After 24 h of biofilm formation, the supernatant was removed and supplemented RPMI was added in the absence (positive control) or presence of the selected compounds (8 - ¼ x MIC). An additional incubation of 24 h at 37°C was performed to evaluate the anti-biofilm activity. Evaluation of both biofilm formation and preformed biofilms was carried out using three chromogenic assays as previously described [25–27]: crystal violet, safranin, and XTT assays were used to analyze the overall biomass, extracellular matrix, and metabolic activity, respectively.

### Analysis of fungal cell alterations

Alterations of *S*. *aurantiacum* cells caused by alexidine, amorolfine and olorofim were analyzed using fluorescent staining. Oxidative stress, mitochondrial membrane potential, and the presence of nucleic acid, mannose residues and chitin were evaluated using 2’,7’–dichlorofluorescein diacetate (DCFH-DA) (Sigma-Aldrich, MO, USA), JC-1 probe (ThermoFisher, Waltham, MA, USA), Sytox Blue (ThermoFisher, Waltham, MA, USA), concanavalin A (Sigma-Aldrich, MO, USA), calcofluor white (Sigma-Aldrich, MO, USA) and Nile Red (Sigma-Aldrich, MO, USA), respectively. Cells were grown in the absence (positive control) or in the presence of 0.5 x MIC of alexidine, amorolfine or olorofim for 48 h at 37°C. Then, cells were stained for 1 h at 37°C under protection from light with 50 μg/mL of DCFH-DA, 10 μg/mL of JC-1, 20 μM of Sytox Blue, 25 μg/mL of concanavalin A or 25 μg/mL of calcofluor white, and 2 mg/mL of Nile Red [28, 29]. Samples were washed three times to remove residual dye and suspended in PBS. Fluorescence intensity was measured using the SpectraMax 340 microplate reader (Molecular Devices, CA, USA) at the following conditions: DCFH-DA at 492 nm (excitation) and 517 nm (emission); JC-1 at 475 nm (excitation) and 529 nm (green fluorescence) or 590 nm (red fluorescence) for the calculation of the red/green fluorescence intensity; Sytox Blue at 444 nm (excitation) and 480 nm (emission); concanavalin A at 495 nm (excitation) and 520 nm (emission); calcofluor white at 350 nm (excitation) and 432 nm (emission); Nile Red at 550 nm (excitation) and 635 nm (emission).

### Scanning electron microscopy (SEM)

*Scedosporium aurantiacum* was grown as planktonic cells, as described above for the previous experiments, or as biofilm in the presence of a sterile fragment of catheter on the bottom of each well. For biofilm formation, after 24 h the supernatant was removed and RPMI 1640 medium supplemented with MOPS, 2% glucose, and 20% fetal bovine serum (FBS, Gibco, Waltham, MA, USA) was added in the absence (positive control) or in the presence of 4x MIC of alexidine, amorolfine or olorofim. After additional 24 h incubation, fragments of the catheter containing adherent biofilm were collected, washed in sterile PBS, and processed for scanning electron microscopy according to Rollin-Pinheiro and colleagues (2017) [14].

Briefly, both planktonic and biofilm samples were processed for microscopy as follows: i. fixation in 2.5% glutaraldehyde and 4% formaldehyde, in 0.1 M cacodylate buffer, for 30 min at room temperature; ii. post-fixation in 1% osmium tetroxide in 0.1 M cacodylate buffer containing 1.25% potassium ferrocyanide for 30 min; iii. dehydration in a graded ethanol series (30–100%); iv. critical point drying in CO2 (EM CPD300, Leica, German); v. adhesion to aluminum stubs with carbon tape; and vi. coating with gold.

Images were obtained with FEI Quanta 250 scanning electron microscope (FEI Company, Hillsboro, OR, USA) and processed using Photoshop software (Adobe, San José, CA, USA).

### Antifungal drug synergy assay

Synergistic interactions were evaluated by the checkerboard method according to EUCAST guidelines [30]. *S*. *aurantiacum* conidia (1 x 10^5^/mL) were grown in 96-well plates containing supplemented RPMI in the presence of selected compounds (0.156–10 μM) combined with voriconazole (0.47–30 μM) or caspofungin (0.625–40 μM). After incubation for 72 h at 37°C, MIC was evaluated at 600 nm and cell viability was assessed by the XTT-reduction assay at 490 nm using a spectrophotometer (Bio-Rad, Hercules, CA, USA). An inhibition of at least 80% was defined as a cut-off for minimum inhibitory concentration (MIC). Interactions were determined by the fractional inhibitory concentration index (FICI), which was calculated using the following formula: (MIC combined/MIC drug A alone) + (MIC combined/MIC drug B alone). The results were classified as: synergistic effect, FICI of ≤0.5; no effect, FICI of >0.5–4.0; antagonistic effect, FICI of >4.0 [31]. Bliss independence model was performed according to Meletiadis and colleagues and Zhao and colleagues [32, 33]. The following formula was used to assess the drug interaction: Eexp = Ea + Eb − Ea × Eb, in which Eexp is the expected efficacy of drug combination, Ea is the efficacy of drug A (Alexidine, amorolfine or olorofim), and Eb is the efficacy of drug B (voriconazole or caspofungin). The results were classified as: synergistic effect, Eobs > Eexp; indifference, Eobs = Eexp; antagonistic effect, Eobs < Eexp.

### Cytotoxicity assay

Cytotoxicity assay of alexidine, amorolfine and olorofim was performed using three cell lineages: RAW 264.7, a murine macrophage culture; A549, a human adenocarcinoma cell line of alveolar basal epithelial cells, and HaCaT, a spontaneously transformed aneuploid immortal keratinocyte cell line from adult human skin. Cells were grown in DMEM (Dulbecco’s Modified Eagle’s Medium, Sigma-Aldrich, MO, USA) supplemented with 10% fetal bovine serum and transferred to 96-well plates to form cell monolayers during an incubation of 24 h at 37°C in 5% CO_2_ atmosphere. Afterwards, cell monolayers were incubated for 48 h at 37°C in 5% CO_2_ in the presence of serially diluted concentrations (0.3–50 μM) of each compound. Then, cell viability was measured using the neutral red (NR) assay and quantified in a spectrophotometer at 595 nm (SpectraMax^®^ i3x, Molecular Devices^®^, San José, CA, EUA) [29, 34].

### Statistical analyses

All experiments were performed in triplicate, in three independent experimental sets. Statistical analyses were performed using GraphPad Prism v5.00 for Windows (GraphPad Software, San Diego, CA, USA). The nonparametric Kruskal–Wallis one-way analysis of variance was used to compare the observed differences among the groups, and individual comparisons of the groups were performed using a Bonferroni post-test. The 90% or 95% confidence interval was determined in all experiments.

## Results

### Screening of pandemic response box library

**Fig 1:**
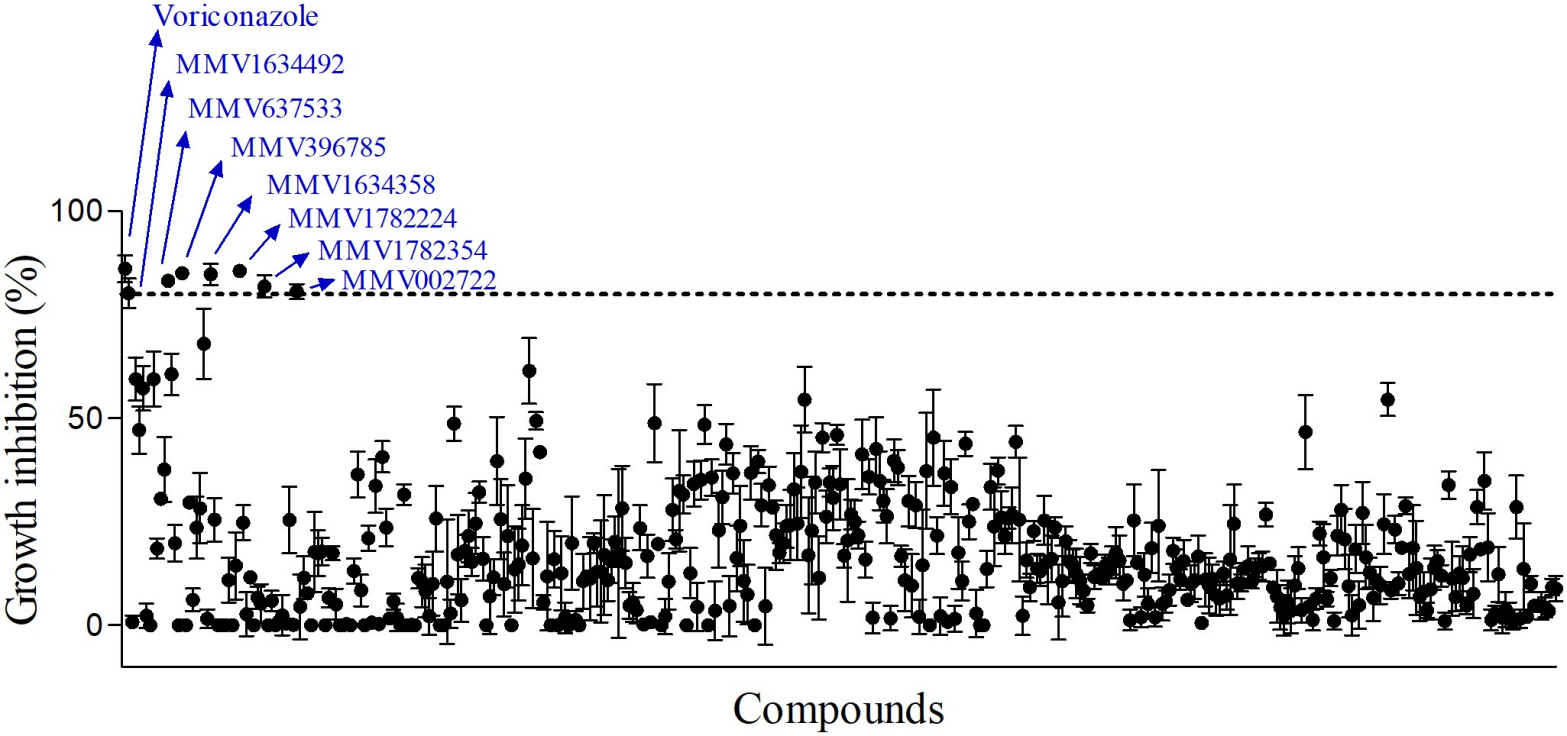
Screening of the Pandemic Response Box^®^ library. A total of 400 compounds were tested against *S*. *aurantiacum* CBS 136046. Fungal growth was quantified after incubation for 72 h by optical density and those presenting at least 80% of inhibition (dotted line) were selected. Voriconazole was used as positive control of inhibition. Experiments were performed in quadruplicate, in three independent experimental sets.

Due to its relevance as a highly virulent and resistant species of *Scedosporium* group [2, 35], *S*. *aurantiacum* was used as reference strain to screen all 400 compounds from the Pandemic Response Box^®^ library (S1 Table). In addition to voriconazole, which was used as control of fungal inhibition, seven compounds inhibited at least 80% of *S*. *aurantiacum* growth and thus were found to display antifungal activity at 5 μM (Fig 1). Among them, eberconazole, ketoconazole, luliconazole and miconazole were identified as members of azole compounds, a known class of antifungal drugs (Table 1). The other three molecules were: alexidine, an antimicrobial drug from the class of bis-biguanide; amorolfine, an antifungal molecule from the class of the morpholine; and olorofim, a molecule from the class of orotomide that shows antifungal properties (Table 1). Several other compounds also inhibited fungal growth at 50–80%, which were identified at antifungal, antibacterial and antiviral molecules (Table 1). However, we decided to focus on compounds that inhibited at least 80% of *S*. *aurantiacum* growth.

**Table 1:** Identification of the selected compounds from the screening of the Pandemic Response Box^®^ library.

Compound code	% of inhibition	Name	Activity/Use
MMV1782224	85.6	Luliconazole	Azole antifungal
MMV396785	85.0	Alexidine	Biguanide antimicrobial
MMV1634358	84.7	Amorolfine	Morpholine antifungal
MMV637533	83.2	Ketoconazole	Azole antifungal
MMV1782354	81.8	Olorofim	Orotomide antifungal
MMV002722	80.6	Miconazole	Azole antifungal
MMV1634492	80.2	Eberconazole	Azole antifungal
MMV1634493	68.0	Abafungin	Echinocandin antifungal
MMV687273	61.4	SQ109	New anti-tuberculosis drug
MMV1634362	60.6	Ravuconazole	Azole antifungal
MMV1634494	59.5	Isavuconazole	Azole antifungal
MMV637528	57.2	Itraconazole	Azole antifungal
MMV1634399	54.5	Non-commercial drug	Antibacterial properties
MMV1580482	54.5	URMC-099-C	Antiviral drug

Considering that the class of azoles is known to be active against *Scedosporium* and *Lomentospora* species and that one azole drug (voriconazole) is the first choice for the treatment of scedosporiosis and lomentosporiosis, we proceed the investigation with alexidine, amorolfine and olorofim, which belong to different classes of molecules.

### Minimum inhibitory concentration (MIC) of alexidine, amorolfine and olorofim against different *Scedosporium* and *Lomentospora* species

**Fig 2:**
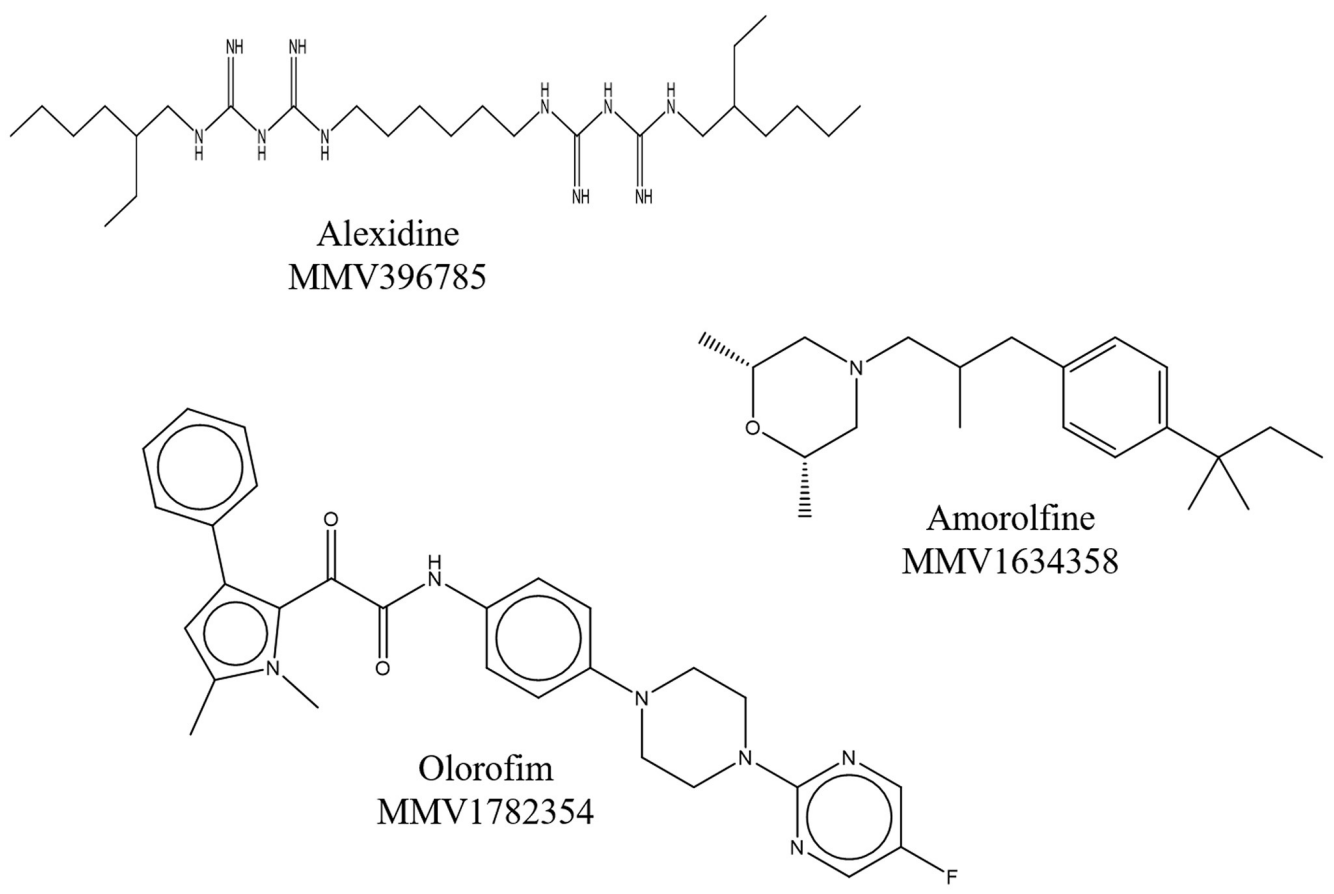
Molecular structures of the compounds with activity against *Scedosporium* and *Lomentospora* species. The compounds present in MMV Pandemic Response Box^®^ library are MMV396785 (Alexidine), MMV1634358 (Amorolfine) and MMV1782354 (Olorofim).

Since alexidine, amorolfine and olorofim are not the usual drugs for treatment of *Scedosporium* and *Lomentospora* infections, they were selected to further investigate their effects on these fungal cells (Fig 2). Considering that the screening of the library was performed using only one concentration of each compound (5 μM), the MIC of alexidine, amorolfine and olorofim was evaluated using the antifungal susceptibility test, and voriconazole as a reference drug. The assay was performed not only against *S*. *aurantiacum*, but also against other clinically relevant species, such as *S*. *boydii*, *S*. *apiospermum*, *S*. *dehoogii*, and *L*. *prolificans*.

Alexidine displayed MIC values varying from 1.25 to 2.5 μM, whereas fungal viability inhibition (measured by the XTT-reduction method that evaluates the metabolic activity) was observed at 1.25, 2.5 and 5 μM of this compound (Table 2). For amorolfine, MIC values were 2.5 μM for *S*. *aurantiacum* and 5 μM for all the other species, whereas inhibition of fungal viability was observed at 5 μM for *S*. *aurantiacum*, 10 μM for *L*. *prolificans* and > 10 μM for all the other species (Table 2). Olorofim MIC values varied from 0.15 μM for *S*. *boydii* to 1.25 μM for *S*. *aurantiacum* and *L*. *prolificans*. Viability inhibition ranged from 0.15–1.25 μM (Table 2). Voriconazole was used as reference drug and, interestingly, olorofim presented MIC values lower than voriconazole for all species. Alexidine and amorolfine also displayed lower MIC compared to voriconazole for *L*. *prolificans* (Table 2).

**Table 2:** Minimum inhibitory concentration of alexidine, amorolfine, olorofim and voriconazole against several *Scedosporium* and *Lomentospora* species.

	Alexidine (μM)		Amorolfine (μM)		Olorofim (μM)		Voriconazole (μM)	
	Growth inhibition	Viability inhibition	Growth inhibition	Viability inhibition	Growth inhibition	Viability inhibition	Growth inhibition	Viability inhibition
***S*. *aurantiacum***	2.5	5	2.5	5	1.25	1.25	1.78	1.78
***S*. *boydii***	2.5	5	5	> 10	0.15	0.15	0.89	1.78
***S*. *apiospermum***	2.5	2.5	5	> 10	0.31	0.62	3.57	3.57
***S*. *dehoogii***	1.25	1.25	5	> 10	0.31	0.31	3.57	3.57
***L*. *prolificans***	1.25	1.25	5	10	1.25	1.25	60	60

Experiments were performed in triplicate, in three independent experimental sets.

### Effect of alexidine, amorolfine and olorofim on fungal biofilms

**Fig 3:**
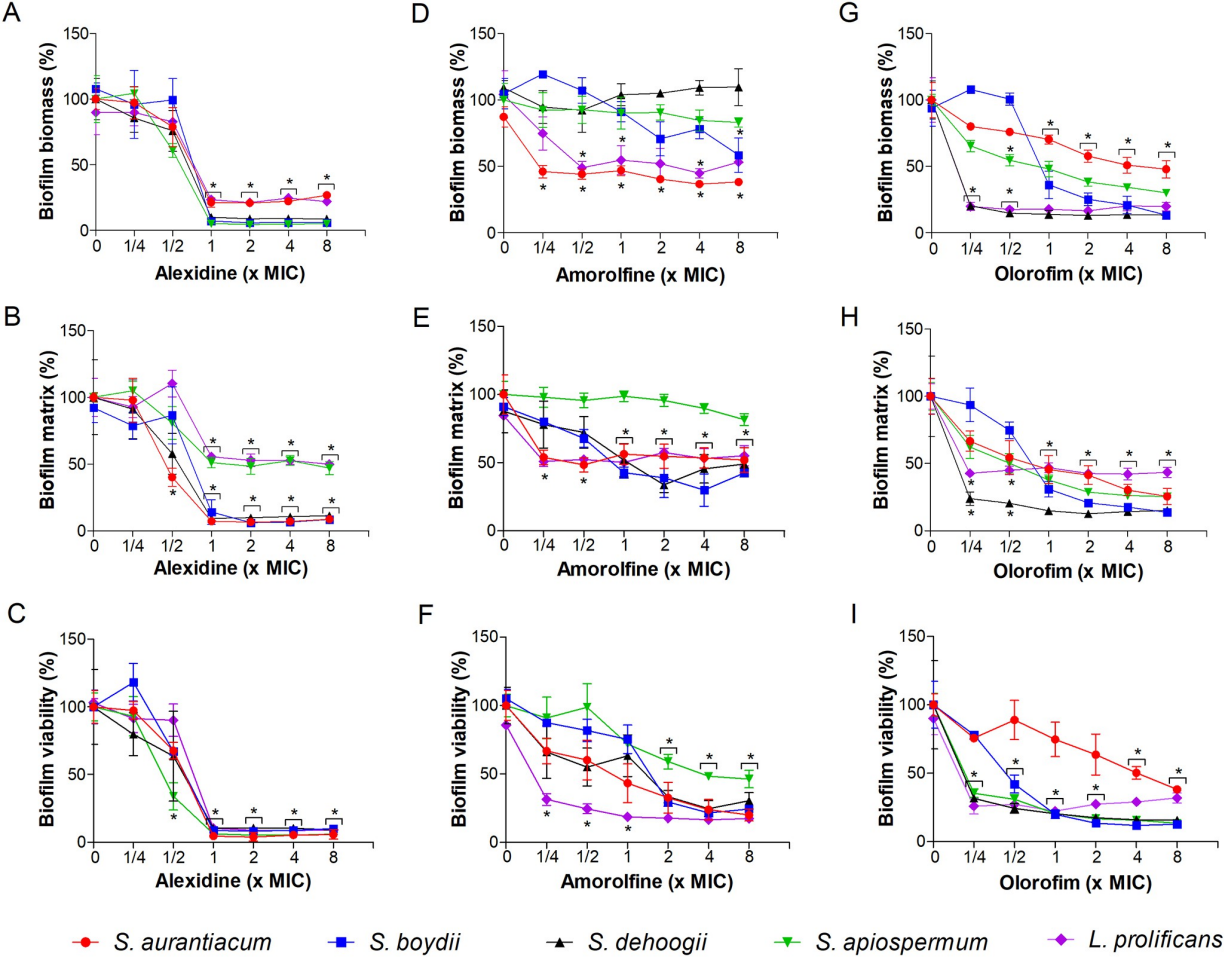
Effect of alexidine, amorolfine and olorofim on biofilm formation of *Scedosporium* and *Lomentospora* species. Fungal cells were adhered on the polystyrene surface for 1.5 h and then different concentrations of alexidine, amorolfine or olorofim were added. Fungal biomass (A, D and G), extracellular matrix (B, E and H) and viability (C, F and I) were measured using crystal violet, safranin and XTT-reduction assay, respectively. * *p* < 0.01, compared to zero (absence of drug) for each species. Experiments were performed in quadruplicate, in three independent experimental sets.

The activity of alexidine, amorolfine and olorofim against *Scedosporium* and *Lomentospora* biofilms was evaluated. Biofilm formation and preformed biofilms were assessed by determining three parameters: fungal biomass, extracellular matrix and cell viability (Tables 1–3 in S2 Table and Tables 1–3 in S3 Table). Regarding biofilm formation, alexidine reduced at least 70% of fungal biomass, extracellular matrix and biofilm viability in all fungal species when MIC or higher concentrations were used (Fig 3A–3C). Amorolfine led to a decrease of approximately 50% of fungal biomass for *S*. *aurantiacum*, *S*. *boydii* and *L*. *prolificans*, especially at 4x MIC. However, no effect was observed for *S*. *apiospermum* and *S*. *dehoogii* (Fig 3D). The extracellular matrix was also reduced by 50% at MIC and higher concentrations, except for *S*. *apiospermum* (Fig 3E). Biofilm viability was decreased by more than 50% for all five species, especially at 2x and 4x MIC of amorolfine (Fig 3F). For olorofim, a decrease of less than 50% of fungal biomass was observed in the presence of 2x, 4x and 8x MIC for all fungi, with the exception of *S*. *aurantiacum*, which in the presence of 8x MIC reached around 50% of the biomass (Fig 3G). At least half of the extracellular matrix was reduced in all species when 1x MIC or higher concentrations were used (Fig 3H). Biofilm viability decreased about 50% in for all five fungi at 1x MIC, except for *S*. *aurantiacum*, where viability decreased 50% at 4x MIC (Fig 3I).

**Fig 4:**
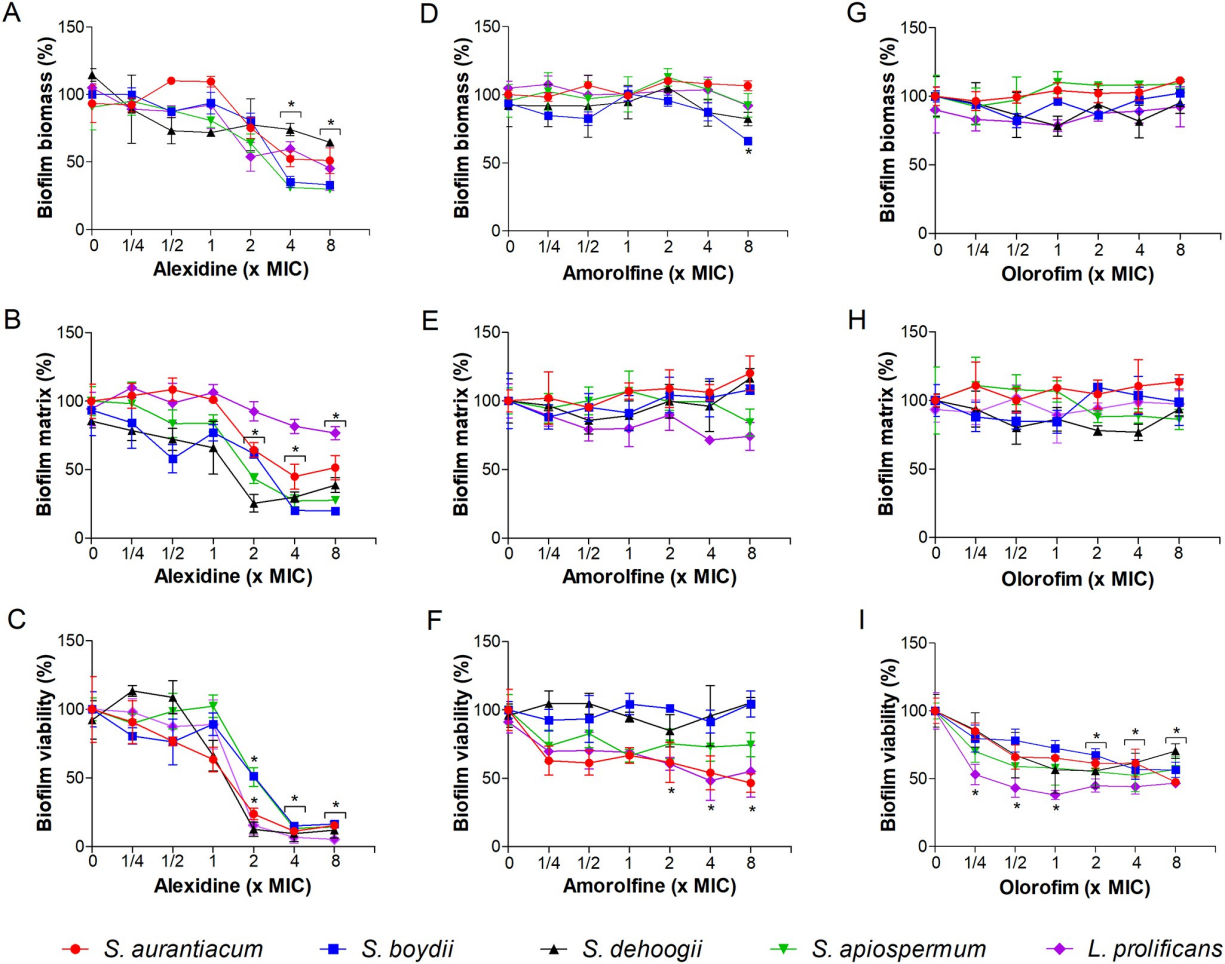
Effect of alexidine, amorolfine and olorofim on preformed biofilms of *Scedosporium* and *Lomentospora* species. Fungal biofilm was obtained in RPMI 1640 medium for 24 h and then treated with different concentrations of alexidine, amorolfine or olorofim for an additional 24 h incubation. Fungal biomass (A, D and G), extracellular matrix (B, E and H) and viability (C, F and I) were measured using crystal violet and safranin stains, and XTT-reduction assay, respectively. * *p* < 0.01, compared to zero (absence of drug) for each species. Experiments were performed in triplicate, in three independent experimental sets.

The evaluation of preformed biofilm revealed that alexidine affected biomass, extracellular matrix and viability when 4x and 8x MIC were used, except for *S*. *dehoogii* and *L*. *prolificans*, in which only biofilm viability was reduced (Fig 4A–4C). Amorolfine did not show activity against preformed biofilm for any of the five species, although a reduction of around 50% of fungal viability was observed for *S*. *aurantiacum* and *L*. *prolificans* when 4x and 8x MIC were used (Fig 4D–4F). Olorofim was not active against preformed biomass and extracellular matrix, but a 50% decrease of viability was observed at 4x and 8x MIC for all five fungi (Fig 4G–4I).

### Alterations on *S*. *aurantiacum* cells caused by alexidine, amorolfine and olorofim

For the evaluation of cell alterations induced by alexidine, amorolfine and olorofim, we performed scanning electron microscopy to analyze fungal morphology of *S*. *aurantiacum* (used as a reference strain) in the presence of the three compounds (S4 Table). Fluorescent probes were used to investigate the alterations of certain cell parameters: DCFDA was used to measure ROS production, JC-1 to assess mitochondrial membrane polarization, Sytox Blue to stain DNA, concanavalin A to detect mannose residues, calcofluor white to analyze chitin content and Nile Red to quantify neutral lipids. Untreated cells were used as a control.

**Fig 5:**
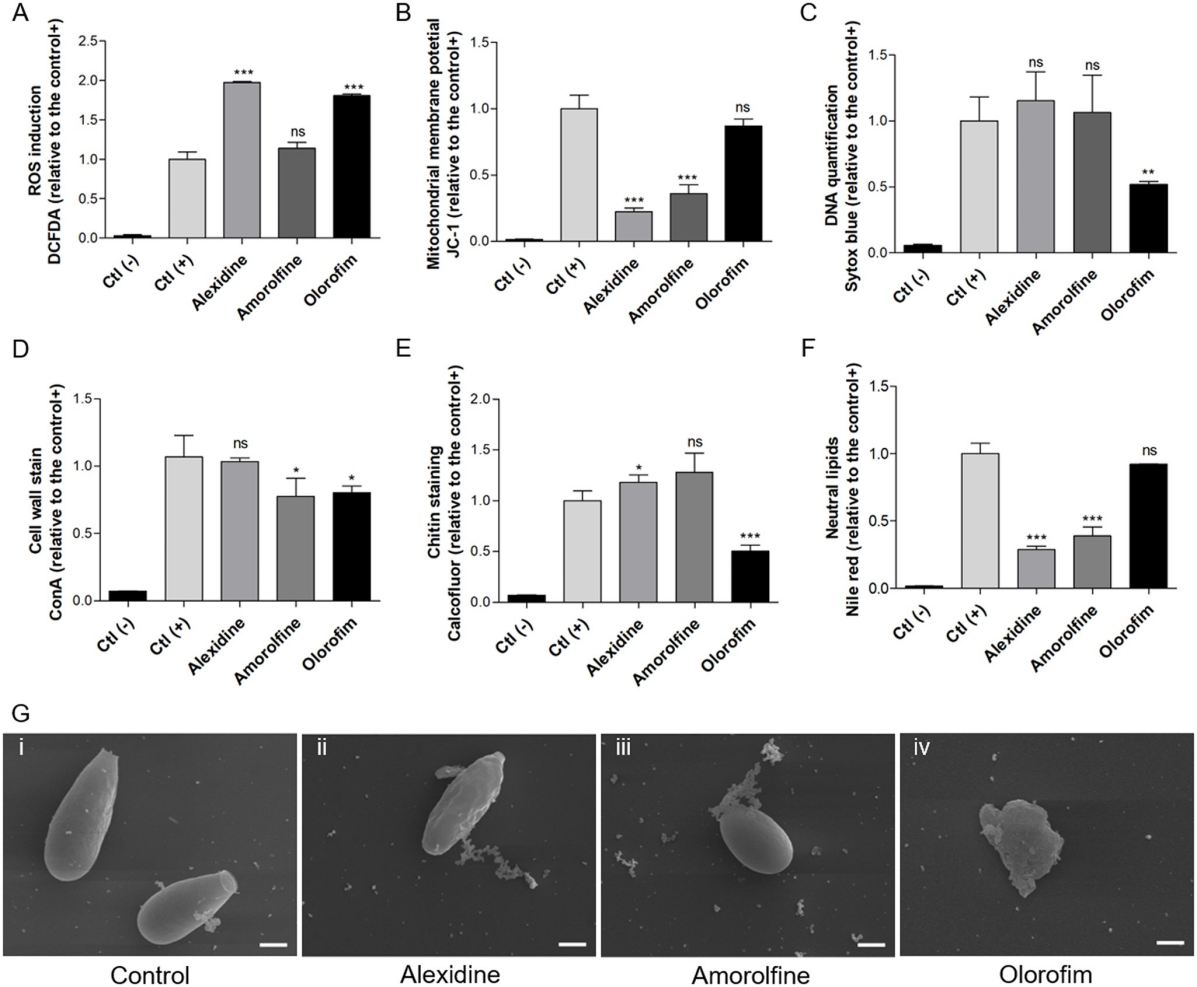
The effect of alexidine, amorolfine and olorofim on *S*. *aurantiacum* morphology and cell parameters. Cells were grown in the presence of ½ x MIC of alexidine, amorolfine or olorofim for 48 h at 37°C. Oxidative stress was measured by DCFH-DA (A). Mitochondrial membrane polarization was measured by JC-1 (B). Intracellular DNA quantification was analyzed by Sytox blue staining (C). Concanavalin A was used to evaluate mannose residues (D). Chitin content was analyzed by calcofluor white (E). Neutral lipids were quantified using Nile Red stain (F). Scanning electron microscopy was performed following the same treatment conditions (G). Ctl (-), a negative control that represents cells in the absence of fluorescent stain. Ctl (+) a positive control that represents cells stained with fluorescent stain, but without drug treatment. Results of treated cells are represented as a relative expression compared to Ctl (+), in other words how many times the fluorescence intensity is observed compared to Ctl (+). * *p* < 0.05; ** *p* < 0.01; *** *p* <0.001; ns = not significant. Experiments were performed in quadruplicate, in three independent experimental sets.

Alexidine and olorofim increased ROS production 2-fold compared to the control, whereas amorolfine led to a slight induction of DCFDA staining (Fig 5A), suggesting that the treatment with all three compounds results in oxidative stress on *S*. *aurantiacum* cells. Alexidine and amorolfine led to mitochondrial membrane depolarization, indicating that these drugs damage fungal mitochondria (Fig 5B). DNA synthesis was not affected by alexidine and amorolfine, but it was 50% reduced when cells were treated with olorofim, suggesting that this compound interferes in the duplication of fungal genetic material (Fig 5C).

Alexidine did not affect concanavalin A staining, but the chitin content was increased as calcofluor white staining was higher compared to the control (Fig 5D and 5E). It might be a compensatory effect, because Nile Red staining revealed that alexidine decreased neutral lipid content (Fig 5F). Corroborating with these observations, SEM analysis showed that *S*. *aurantiacum* surface seems to be rougher compared to the control (Fig 5G), possibly as result of changes in fungal cell wall and plasma membrane, although more experiments are needed for confirmation. Cells treated with amorolfine presented a decrease of mannose residues (Fig 5D) and neutral lipid content (Fig 5F), suggesting that this drug also led to a deregulation of the fungal surface components. However, SEM did not demonstrate relevant alterations on the fungal surface when cells were treated with amorolfine (Fig 5G). Treatment with olorofim led to a decrease of mannose residues and chitin content, indicating an alteration in the composition of fungal cell wall (Fig 5D and 5E). In addition, SEM images revealed modifications of the surface and morphology of *S*. *aurantiacum* (Fig 5G).

### Scanning electron microscopy to evaluate morphological alterations caused by alexidine, amorolfine and olorofim on *S*. *aurantiacum* biofilm

Considering that alexidine, amorolfine and olorofim displayed antifungal activity against planktonic cells and biofilms, the morphology of *S*. *aurantiacum* biofilm grown on a catheter surface was evaluated using scanning electron microscopy (SEM). *S*. *aurantiacum* was used as a reference strain because it was the species used for the screening of the Pandemic Response Box^®^ library. The concentration of the drugs used on *S*. *aurantiacum* preformed biofilm was equivalent to 4x MIC.

**Fig 6:**
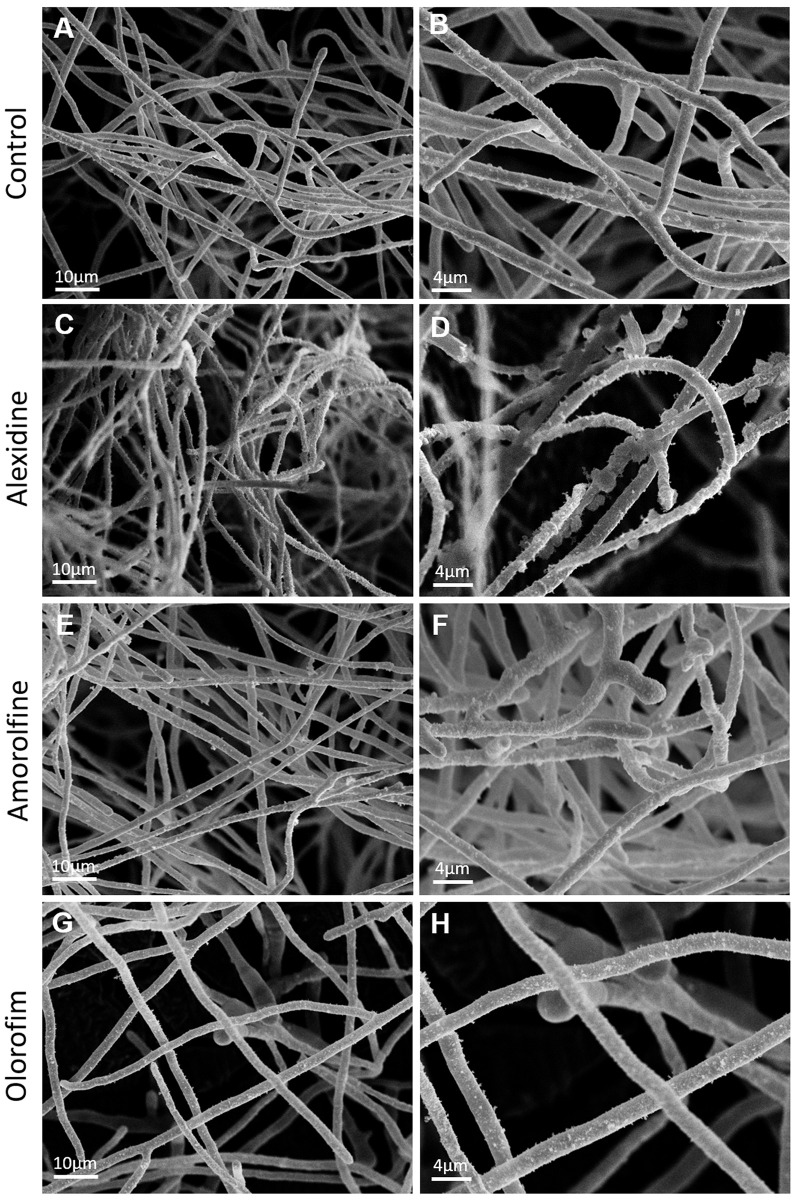
Morphological alterations on *S*. *aurantiacum* biofilm by scanning electron microscopy. *S*. *aurantiacum* CBS 136046 preformed biofilm was incubated in the presence of alexidine, amorolfine or olorofim for 24 h at 37°C. Untreated cells were used as a control. Bars: 10 μm (A, C, E and G) e 4 μm (B, D, F and H).

Treatment with alexidine, the most effective drug against preformed biofilms, revealed bleb-like structures along the entire hyphal surfaces (Fig 6C and 6D), when compared to untreated *S*. *aurantiacum* biofilm that did not show such alterations (Fig 6A and 6B). Amorolfine and olorofim showed no morphological alterations as they were not highly effective against preformed biofilms (Fig 6E–6H).

### Drug Interactions for alexidine, amorolfine and olorofim with voriconazole and caspofungin

Since none of these compounds are antifungal drugs usually employed in clinical settings, we decided to investigate the interaction properties of alexidine, amorolfine and olorofim with some known antifungal drugs. A checkerboard analysis was performed and, once again, *S*. *aurantiacum* was used as a representative species. Drug interactions were analyzed between alexidine, amorolfine or olorofim with voriconazole or caspofungin.

The analyses performed using the checkerboard method and the calculation of FIC index revealed that none of the drug combinations resulted in synergistic interaction, although the MIC of caspofungin reduced when in combination with amorolfine and olorofim (Table 3). However, using the Bliss independence method, alexidine and amorolfine presented a synergistic effect when combined either with voriconazole or caspofungin (Table 4). Olorofim displayed a synergistic interaction with caspofungin, but an antagonistic interaction with voriconazole (Table 4).

**Table 3:** Antifungal activity of alexidine, amorolfine, olorofim, voriconazole and caspofungin—alone and in combinations according to fractional inhibitory concentration index (FICI)—against *S*. *aurantiacum* CBS 136046.

MIC_80_ alone (μM)		MIC_80_ combined (μM)		FICI
**Alexidine**	2.5	**Alex/Vorico**	2.5/1.78	2.0 (no effect)
**Amorolfine**	2.5	**Alex/Caspo**	2.5/20	2.0 (no effect)
**Olorofim**	1.25	**Amo/Vorico**	1.25/1.78	1.5 (no effect)
**Voriconazole**	1.78	**Amo/Caspo**	1.25/10	1.0 (no effect)
**Caspofungin**	20	**Olo/Vorico**	1.25/1.78	2.0 (no effect)
		**Olo/Caspo**	1.25/10	1.5 (no effect)

MIC: Minimal inhibitory concentration. FICI: Fractional Inhibitory Concentration Index. Alex: Alexidine. Amo: Amorolfine. Olo: Olorofim. Vorico: Voriconazole. Caspo: Caspofungin. Experiments were performed in triplicate, in three independent experimental sets.

**Table 4:** Antifungal activity of alexidine, amorolfine, olorofim, voriconazole and caspofungin—alone and in combinations according to the Bliss independence model.

	Efficacy of drugs alone (% of inhibition)		Efficacy of combined drugs					
			Voriconazole			Caspofungin		
	MIC_80_	½ MIC_80_	*E*_obs_	*E*_exp_	Δ*E*, % (interaction)	*E*_obs_	*E*_exp_	Δ*E*, % (interaction)
Alexidine	89.8	7.8	44.4	42.5	1.9 (**S**)	31.8	12.4	19.4 (**S**)
Amorolfine	91.6	49.7	90.2	66.7	23.5 (**S**)	90.8	74.2	16.6 (**S**)
Olorofim	79.4	41.8	31.0	48.1	-17.1 (**A**)	80.2	48.5	31.7 (**S**)
Voriconazole	89.1	33.7	NP	NP	NP	NP	NP	NP
Caspofungin	82.4	11.5	NP	NP	NP	NP	NP	NP

MIC: Minimal inhibitory concentration. *E*obs, observed efficacy in the analysis. *E*exp, expected efficacy according to Bliss calculation. Δ*E*, difference between *E*obs and *E*exp. NP, not performed. S, synergistic interaction. A, antagonistic interaction. Experiments were performed in triplicate, in three independent experimental sets.

### Cytotoxicity of alexidine, amorolfine and olorofim

**Fig 7:**
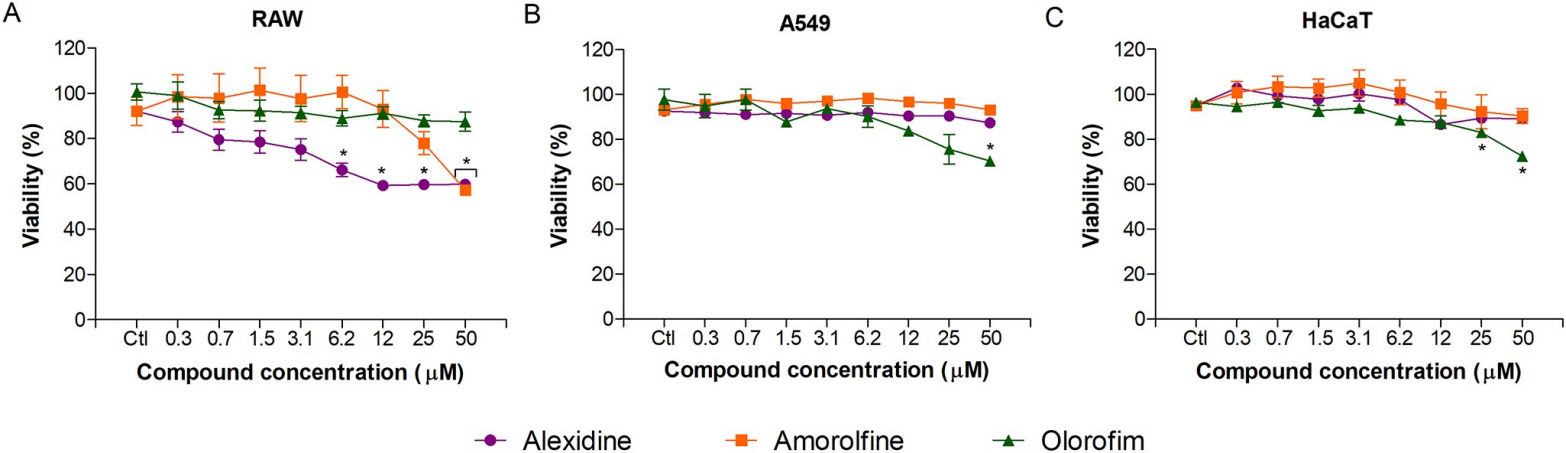
Cytotoxicity assay. Monolayers of RAW 264.7, A549 and HaCaT cells were incubated for 24 h in the presence of 0.3–50 μM of alexidine, amorolfine or olorofim, and cell viability was measured by neutral red method. * *p* < 0.05. Experiments were performed in triplicate, in three independent experimental sets.

Cytotoxicity of the three selected compounds was evaluated using a mouse cell lineage, RAW 264.7, and two human cell lineages, A549 and HaCaT (S5 Table). Alexidine at 6.2 μM reduced more than 30% of RAW 264.7 viability, but a reduction of more than 50% of viability was not observed, even at higher concentrations (Fig 7A). In A549 and HaCaT cell lineages, alexidine did not display any toxic effect even at the highest concentration tested (50 μM) (Fig 7B and 7C). For amorolfine, a 40% reduction of viability was observed for RAW 264.7 cells when 50 μM was used (Fig 7A), but no effect was observed on A549 and HaCaT at any of the concentrations used (Fig 7B and 7C). Olorofim did not display any toxic effect in RAW 264.7 cells even at higher concentrations (Fig 7A) and only 30% of viability reduction was observed for A549 and HaCaT when the highest (50 μM) concentration was used (Fig 7B and 7C).

## Discussion

The present study tested the library of compounds called Pandemic Response Box^®^ against fungal species belonging to *Scedosporium* and *Lomentospora* genera. Due to the emergence of highly virulent isolates that are resistant to antifungal agents, this approach is considered an urgent need [1, 7]. The screening of the Pandemic Response Box^®^ revealed three promising compounds with antifungal activity: alexidine, amorolfine and olorofim.

Alexidine, a member of the bis-biguanide class, is not a conventional antifungal drug, but is used as an antimicrobial agent in solutions for contact lenses and mouthwashes [36]. In our study, alexidine inhibited the growth of all tested species. Mamouei and colleagues screened the New Prestwick Chemical Library and demonstrated that alexidine inhibits at least 50% of the growth of several *Candida* species (including *C*. *albicans* and *C*. *auris*), *Cryptococcus neoformans*, *Aspergillus fumigatus*, several species from the Mucorales order, and *S*. *apiospermum* [37]. Yousfi and colleagues reported the activity of alexidine against other species of filamentous fungi including *A*. *calidoustus*, *Fusarium solani*, *F*. *oxysporum* as well as *L*. *prolificans* [38]. Together with these previous studies our results provide further indication for a broad-spectrum antifungal activity of alexidine, also acting against other *Scedosporium* species.

Alexidine also reduced biofilm formation. Mamouei and colleagues reported the anti-biofilm potential of alexidine in *A*. *fumigatus*, *C*. *neoformans* and *Candida* species. Alexidine was also effective against preformed biofilms from *C*. *albicans* in *in vitro* an *in vivo* catheter model [37]. However, the effect of alexidine on *Scedosporium* and *Lomentospora* biofilms has never been evaluated and our data demonstrated that alexidine was the most effective drug against biofilms of these species when compared to the other selected drugs.

The mechanism of action of alexidine in fungi is unknown and our study is the first to demonstrate alterations in fungal cell wall caused by this compound. In bacteria, positively charged alexidine is attracted to the negatively charged bacterial cell wall and induces lipid-phase separation and lipid raft formation in bacterial membranes [36]. The fungal cell wall is also negatively charged, which might hint on a similar action for alexidine. Similarly to what we observed in *Scedosporium* and *Lomentospora* species, previous reports showed that in some cancer cell lines alexidine affects membrane permeability and induces mitochondrial damage by targeting mitochondrial phosphatase PTPMT1 which might correlate with its effect in other eukaryotic cells like fungi [39, 40].

The combinations of alexidine with voriconazole and caspofungin were considered synergistic using the Bliss method. In *C*. *albicans*, alexidine also enhanced the effect of an azole agent, fluconazole [37]. However, little is known about the interaction between alexidine and antifungal drugs, and our study is the first to report it in filamentous fungi. Thus, more studies are needed to clarify the effect of these combinations.

Alexidine cytotoxicity has already been described in the literature. Mamouei and colleagues observed that alexidine damaged HUVEC, an endothelial cell line, and A549 cells at 14.7 μg/ml (28.89 μM) [37], but a comparison with our results is difficult due to differences in the methodologies, such as the reading technique used to measure cytotoxicity. Nevertheless, only concentrations higher than the MICs for fungi might damage host cells. Although our study shows cytotoxic data using three different cell lineages, more studies are needed to evaluate the safety of alexidine, especially *in vivo*.

Amorolfine, from the morpholine class, is a known antifungal drug used for the treatment of onychomycosis and acts by inhibiting two enzymes from the ergosterol synthesis pathway, delta-14-reductase and delta-7,8-isomerase. Amorolfine is approved for onychomycosis treatment and its effectiveness against other fungal pathogens including yeasts and dimorphic fungi has been demonstrated [41]. It also acts against some filamentous fungi like *Scopulariopsis*, *Alternaria* and *Fusarium*, but *A*. *fumigatus* showed low susceptibility [41]. Although amorolfine is already used in clinical settings for the treatment of dermatophytic infections, this drug is still not recommended for the treatment of invasive fungal infections since it is rapidly metabolized by the human body [41]. Therefore, it does not seem adequate for invasive scedosporiosis or lomentosporiosis but could be effective in localized infections, although *in vivo* studies are needed to investigate its efficacy in this model [42]. In our study, this compound inhibited the growth of all tested species, but failed to reduce metabolic activity against *S*. *boydii*, *S*. *apiospermum* and *S*. *dehoogii* at the tested concentrations. Regarding its effect on biofilms, there are no reports that show its activity against *Scedosporium* and *Lomentospora* biofilms, but our data demonstrated that amorolfine decrease its formation although preformed biofilms were more resistant.

Amorolfine acts in fungi as inhibitor of two steps of the ergosterol biosynthesis pathway. Thus, the alteration of the ergosterol content in fungal cells might explain the reduction in Nile Red staining observed in the present study. Disruption in ergosterol synthesis impairs several cellular processes since ergosterol is also present in the membranes of organelles including mitochondria [43].

Liu and colleagues reported the synergistic effect of amorolfine and voriconazole against several strains of *F*. *solani* and *F*. *oxysporum* both *in vitro* as well as in a *Galleria mellonella* model [44], similarly to what we observed in *Scedosporium* and *Lomentospora* species when amorolfine were combined with voriconazole or caspofungin.

Olorofim is the first member of a new class of antifungals called orotomides and it inhibits dihydroorotate dehydrogenase (DHODH), disrupting *de novo* pyrimidine synthesis [45]. It showed potent *in vitro* activity against different species of filamentous and dimorphic fungi. However, it is not effective against clinically important yeasts (such as *Candida* and *Cryptococcus*) and fungi from the order *Mucorales* due to structural differences in the targeted enzyme [45–47]. Our results are in agreement with other studies that demonstrated that olorofim is highly active *in vitro* against *Scedosporium* and *Lomentospora* [48]. On the other hand, although it also reduced biofilm formation, low activity was observed against preformed biofilms.

Olorofim mechanism of action affects uridine-50-monophosphate (UMP) and uridine-50-triphosphate (UTP) production and consequently impairing RNA/DNA synthesis [45, 49]. UTP is also necessary for the formation of UDP-glucose and UDP-*N*-acetyl-glucosamine that are building blocks for several glycoconjugates of the fungal cell wall [50]. It was already observed in *A*. *fumigatus* that olorofim significantly influences cell dynamics by reducing mitosis and β-1,3-glucan content in hyphal tips [51]. These consequences could explain the cell alterations we observed in the present study. Additionally, unlike our observation for *Scedosporium* and *Lomentospora* species, olorofim treatment of *A*. *fumigatus* increased cell wall chitin content which was hypothesized as being a stress response to prevent cell lysis [51].

Interactions between olorofim and antifungal drugs revealed antagonist effect with voriconazol. Antagonism between olorofim and azoles, specifically itraconazole and voriconazole, has also been previously reported for *A*. *fumigatus* [52]. However, the mechanism that leads to antagonism between olorofim and azoles remains unclear and further studies are needed for its elucidation. In addition, these observations are important since voriconazole is the first choice to treat scedosporiosis. Therefore, an understanding of the interaction between olorofim and voriconazole in clinical settings is an urgent need.

*In vivo* tests already showed that olorofim is a promising drug for the treatment of aspergillosis, scedosporiosis and lomentosporiosis [53, 54]. The U.S. Food and Drug Administration (FDA) granted olorofim the designation of breakthrough therapy in 2019 and the status of orphan drug in 2020. The European Medicines Agency Committee for Orphan Medicinal Products also granted the orphan drug status to olorofim for treatment of invasive aspergillosis and scedosporiosis. Currently, a phase 2 clinical trial of olorofim is being conducted for treatment of invasive fungal diseases including aspergillosis, scedosporiosis and lomentosporiosis [55].

*Scedosporium* and *Lomentospora* species are considered rare and neglected fungi, so little is known in the literature about the mechanism of action of promising compounds. Consequently, the identification and the description of new compounds with anti-*Scedosporium* and anti-*Lomentospora* activity is an urgent need. In our study, three drugs were selected by screening the Pandemic Response Box^®^, which represents a contribution in the literature to point out some potent candidates for the treatment of *Scedosporium* and *Lomentospora* infections.

## Supporting information

S1 Table Pandemic Response Box library screening data. A total of 400 compounds were tested against *S*. *aurantiacum* CBS 136046.
(XLSX)

S2 Table Biofilm formation. Data of alexidine (Table 1), amorolfine (Table 2) or olorofim (Table 3) effect on biofilm formation of *Scedosporium* and *Lomentospora* species.
(XLSX)

S3 Table Preformed biofilm. Data of alexidine (Table 1), amorolfine (Table 2) or olorofim (Table 3) effect on biofilm formation of *Scedosporium* and *Lomentospora* species.
(XLSX)

S4 Table Alterations on *S*. *aurantiacum* cells. Data of the alexidine, amorolfine and olorofim effect in *S*. *aurantiacum* cells analyzed using fluorescent staining.
(XLSX)

S5 Table Cytotoxicity assay. Data of alexidine, amorolfine and olorofim effect on RAW, A549 and HaCaT cell lines.
(XLSX)

## References

[1] Cortez KJ, Roilides E, Quiroz-Telles F, Meletiadis J, Antachopoulos C, Knudsen T, et al. Infections caused by Scedosporium spp. Clin Microbiol Rev. 2008;21(1):157–97. doi: 10.1128/CMR.00039-07 . 18202441 PMC2223844

[2] Gilgado F, Cano J, Gené J, Serena C, Guarro J. Different virulence of the species of the Pseudallescheria boydii complex. Med Mycol. 2009;47(4):371–4. Epub 20080709. doi: 10.1080/13693780802256539 . 18651312

[3] Luplertlop N. Pseudallescheria/Scedosporium complex species: From saprobic to pathogenic fungus. J Mycol Med. 2018;28(2):249–56. Epub 20180319. doi: 10.1016/j.mycmed.2018.02.015 . 29567285

[4] Engel TGP, Slabbers L, de Jong C, Melchers WJG, Hagen F, Verweij PE, et al. Prevalence and diversity of filamentous fungi in the airways of cystic fibrosis patients—A Dutch, multicentre study. J Cyst Fibros. 2019;18(2):221–6. Epub 20181201. doi: 10.1016/j.jcf.2018.11.012 . 30514613

[5] Cowen LE. The evolution of fungal drug resistance: modulating the trajectory from genotype to phenotype. Nat Rev Microbiol. 2008;6(3):187–98. doi: 10.1038/nrmicro1835 . 18246082

[6] Rollin-Pinheiro R, Singh A, Barreto-Bergter E, Del Poeta M. Sphingolipids as targets for treatment of fungal infections. Future Med Chem. 2016;8(12):1469–84. Epub 20160809. doi: 10.4155/fmc-2016-0053 . 27502288 PMC5558548

[7] Hoenigl M, Salmanton-García J, Walsh TJ, Nucci M, Neoh CF, Jenks JD, et al. Global guideline for the diagnosis and management of rare mould infections: an initiative of the European Confederation of Medical Mycology in cooperation with the International Society for Human and Animal Mycology and the American Society for Microbiology. Lancet Infect Dis. 2021;21(8):e246–e57. Epub 20210216. doi: 10.1016/S1473-3099(20)30784-2 . 33606997

[8] Lackner M, de Hoog GS, Verweij PE, Najafzadeh MJ, Curfs-Breuker I, Klaassen CH, et al. Species-specific antifungal susceptibility patterns of Scedosporium and Pseudallescheria species. Antimicrob Agents Chemother. 2012;56(5):2635–42. Epub 20120130. doi: 10.1128/AAC.05910-11 . 22290955 PMC3346635

[9] Pellon A, Ramirez-Garcia A, Buldain I, Antoran A, Martin-Souto L, Rementeria A, et al. Pathobiology of Lomentospora prolificans: could this species serve as a model of primary antifungal resistance? Int J Antimicrob Agents. 2018;51(1):10–5. Epub 20170629. doi: 10.1016/j.ijantimicag.2017.06.009 . 28669833

[10] Ramirez-Garcia A, Pellon A, Rementeria A, Buldain I, Barreto-Bergter E, Rollin-Pinheiro R, et al. Scedosporium and Lomentospora: an updated overview of underrated opportunists. Med Mycol. 2018;56(suppl_1):102–25. doi: 10.1093/mmy/myx113 . 29538735

[11] Tortorano AM, Richardson M, Roilides E, van Diepeningen A, Caira M, Munoz P, et al. ESCMID and ECMM joint guidelines on diagnosis and management of hyalohyphomycosis: Fusarium spp., Scedosporium spp. and others. Clin Microbiol Infect. 2014;20 Suppl 3:27–46. doi: 10.1111/1469-0691.12465 . 24548001

[12] Seidel D, Meißner A, Lackner M, Piepenbrock E, Salmanton-García J, Stecher M, et al. Prognostic factors in 264 adults with invasive Scedosporium spp. and Lomentospora prolificans infection reported in the literature and FungiScope(^®^). Crit Rev Microbiol. 2019;45(1):1–21. Epub 20190110. doi: 10.1080/1040841x.2018.1514366 . 30628529

[13] Rodriguez-Tudela JL, Berenguer J, Guarro J, Kantarcioglu AS, Horre R, de Hoog GS, et al. Epidemiology and outcome of Scedosporium prolificans infection, a review of 162 cases. Med Mycol. 2009;47(4):359–70. doi: 10.1080/13693780802524506 . 19031336

[14] Rollin-Pinheiro R, de Meirelles JV, Vila TVM, Fonseca BB, Alves V, Frases S, et al. Biofilm Formation by Pseudallescheria/Scedosporium Species: A Comparative Study. Front Microbiol. 2017;8:1568. Epub 20170818. doi: 10.3389/fmicb.2017.01568 . 28868050 PMC5563321

[15] Veale CGL. Unpacking the Pathogen Box-An Open Source Tool for Fighting Neglected Tropical Disease. ChemMedChem. 2019;14(4):386–453. Epub 20190130. doi: 10.1002/cmdc.201800755 . 30614200

[16] Borba-Santos LP, Vila T, Rozental S. Identification of two potential inhibitors of Sporothrix brasiliensis and Sporothrix schenckii in the Pathogen Box collection. PLoS One. 2020;15(10):e0240658. Epub 20201014. doi: 10.1371/journal.pone.0240658 . 33052959 PMC7556523

[17] Coelho RA, Joffe LS, Alves GM, Figueiredo-Carvalho MHG, Brito-Santos F, Amaral ACF, et al. A screening of the MMV Pathogen Box^®^ reveals new potential antifungal drugs against the etiologic agents of chromoblastomycosis. PLoS One. 2020;15(5):e0229630. Epub 20200513. doi: 10.1371/journal.pone.0229630 . 32401759 PMC7219733

[18] May HC, Yu JJ, Guentzel MN, Chambers JP, Cap AP, Arulanandam BP. Repurposing Auranofin, Ebselen, and PX-12 as Antimicrobial Agents Targeting the Thioredoxin System. Front Microbiol. 2018;9:336. Epub 20180305. doi: 10.3389/fmicb.2018.00336 . 29556223 PMC5844926

[19] Vila T, Lopez-Ribot JL. Screening the Pathogen Box for Identification of Candida albicans Biofilm Inhibitors. Antimicrob Agents Chemother. 2017;61(1). Epub 20161227. doi: 10.1128/AAC.02006-16 . 27795383 PMC5192139

[20] Wall G, Herrera N, Lopez-Ribot JL. Repositionable Compounds with Antifungal Activity against Multidrug Resistant Candida auris Identified in the Medicines for Malaria Venture’s Pathogen Box. J Fungi (Basel). 2019;5(4). Epub 20191001. doi: 10.3390/jof5040092 . 31581540 PMC6958377

[21] de Oliveira HC, Castelli RF, Reis FCG, Samby K, Nosanchuk JD, Alves LR, et al. Screening of the Pandemic Response Box Reveals an Association between Antifungal Effects of MMV1593537 and the Cell Wall of Cryptococcus neoformans, Cryptococcus deuterogattii, and Candida auris. Microbiol Spectr. 2022;10(3):e0060122. Epub 20220426. doi: 10.1128/spectrum.00601-22 . 35471056 PMC9241760

[22] Lim W, Nyuykonge B, Eadie K, Konings M, Smeets J, Fahal A, et al. Screening the pandemic response box identified benzimidazole carbamates, Olorofim and ravuconazole as promising drug candidates for the treatment of eumycetoma. PLoS Negl Trop Dis. 2022;16(2):e0010159. Epub 20220204. doi: 10.1371/journal.pntd.0010159 . 35120131 PMC8815882

[23] Rollin-Pinheiro R, Borba-Santos LP, da Silva Xisto MID, de Castro-Almeida Y, Rochetti VP, Rozental S, et al. Identification of Promising Antifungal Drugs against Scedosporium and Lomentospora Species after Screening of Pathogen Box Library. J Fungi (Basel). 2021;7(10). Epub 20210925. doi: 10.3390/jof7100803 . 34682224 PMC8539698

[24] Taj-Aldeen SJ, Salah H, Al-Hatmi AM, Hamed M, Theelen B, van Diepeningen AD, et al. In vitro resistance of clinical Fusarium species to amphotericin B and voriconazole using the EUCAST antifungal susceptibility method. Diagn Microbiol Infect Dis. 2016;85(4):438–43. Epub 20160512. doi: 10.1016/j.diagmicrobio.2016.05.006 . 27312690

[25] Mello TP, Aor AC, Gonçalves DS, Seabra SH, Branquinha MH, Santos AL. Assessment of biofilm formation by Scedosporium apiospermum, S. aurantiacum, S. minutisporum and Lomentospora prolificans. Biofouling. 2016;32(7):737–49. doi: 10.1080/08927014.2016.1192610 . 27309801

[26] Mello TP, Oliveira SSC, Frasés S, Branquinha MH, Santos ALS. Surface properties, adhesion and biofilm formation on different surfaces by Scedosporium spp. and Lomentospora prolificans. Biofouling. 2018;34(7):800–14. Epub 20181024. doi: 10.1080/08927014.2018.1503652 . 30354689

[27] Rollin-Pinheiro R, Rochetti VP, Xisto M, Liporagi-Lopes LC, Bastos B, Rella A, et al. Sphingolipid biosynthetic pathway is crucial for growth, biofilm formation and membrane integrity of Scedosporium boydii. Future Med Chem. 2019;11(22):2905–17. Epub 20191112. doi: 10.4155/fmc-2019-0186 . 31713454 PMC7270895

[28] de Oliveira EB, Xisto M, Rollin-Pinheiro R, Rochetti VP, Barreto-Bergter E. Peptidorhamnomannans From Scedosporium and Lomentospora Species Display Microbicidal Activity Against Bacteria Commonly Present in Cystic Fibrosis Patients. Front Cell Infect Microbiol. 2020;10:598823. Epub 20201028. doi: 10.3389/fcimb.2020.598823 . 33251161 PMC7673444

[29] Rollin-Pinheiro R, Almeida YC, Rochetti VP, Xisto M, Borba-Santos LP, Rozental S, et al. Miltefosine Against Scedosporium and Lomentospora Species: Antifungal Activity and Its Effects on Fungal Cells. Front Cell Infect Microbiol. 2021;11:698662. Epub 20210723. doi: 10.3389/fcimb.2021.698662 . 34368017 PMC8343104

[30] EUCAST Technical Note on the method for the determination of broth dilution minimum inhibitory concentrations of antifungal agents for conidia-forming moulds. Clin Microbiol Infect. 2008;14(10):982–4. doi: 10.1111/j.1469-0691.2008.02086.x . 18828858

[31] Odds FC. Synergy, antagonism, and what the chequerboard puts between them. J Antimicrob Chemother. 2003;52(1):1. Epub 20030612. doi: 10.1093/jac/dkg301 . 12805255

[32] Meletiadis J, Petraitis V, Petraitiene R, Lin P, Stergiopoulou T, Kelaher AM, et al. Triazole-polyene antagonism in experimental invasive pulmonary aspergillosis: in vitro and in vivo correlation. J Infect Dis. 2006;194(7):1008–18. Epub 20060828. doi: 10.1086/506617 . 16960790

[33] Zhao W, Sachsenmeier K, Zhang L, Sult E, Hollingsworth RE, Yang H. A New Bliss Independence Model to Analyze Drug Combination Data. J Biomol Screen. 2014;19(5):817–21. Epub 20140203. doi: 10.1177/1087057114521867 . 24492921

[34] Borenfreund E, Puerner JA. Toxicity determined in vitro by morphological alterations and neutral red absorption. Toxicol Lett. 1985;24(2–3):119–24. doi: 10.1016/0378-4274(85)90046-3 . 3983963

[35] Harun A, Serena C, Gilgado F, Chen SC, Meyer W. Scedosporium aurantiacum is as virulent as S. prolificans, and shows strain-specific virulence differences, in a mouse model. Med Mycol. 2010;48 Suppl 1:S45–51. doi: 10.3109/13693786.2010.517224 . 21067330

[36] McDonnell G, Russell AD. Antiseptics and disinfectants: activity, action, and resistance. Clin Microbiol Rev. 1999;12(1):147–79. doi: 10.1128/CMR.12.1.147 . 9880479 PMC88911

[37] Mamouei Z, Alqarihi A, Singh S, Xu S, Mansour MK, Ibrahim AS, et al. Alexidine Dihydrochloride Has Broad-Spectrum Activities against Diverse Fungal Pathogens. mSphere. 2018;3(5). Epub 20181031. doi: 10.1128/mSphere.00539-18 . 30381356 PMC6211222

[38] Yousfi H, Ranque S, Cassagne C, Rolain JM, Bittar F. Identification of repositionable drugs with novel antimycotic activity by screening the Prestwick Chemical Library against emerging invasive moulds. J Glob Antimicrob Resist. 2020;21:314–7. Epub 20200128. doi: 10.1016/j.jgar.2020.01.002 . 32004725

[39] Doughty-Shenton D, Joseph JD, Zhang J, Pagliarini DJ, Kim Y, Lu D, et al. Pharmacological targeting of the mitochondrial phosphatase PTPMT1. J Pharmacol Exp Ther. 2010;333(2):584–92. Epub 20100218. doi: 10.1124/jpet.109.163329 . 20167843 PMC2872949

[40] Yip KW, Ito E, Mao X, Au PY, Hedley DW, Mocanu JD, et al. Potential use of alexidine dihydrochloride as an apoptosis-promoting anticancer agent. Mol Cancer Ther. 2006;5(9):2234–40. doi: 10.1158/1535-7163.MCT-06-0134 . 16985057

[41] Haria M, Bryson HM. Amorolfine. A review of its pharmacological properties and therapeutic potential in the treatment of onychomycosis and other superficial fungal infections. Drugs. 1995;49(1):103–20. doi: 10.2165/00003495-199549010-00008 . 7705210

[42] Jachak GR, Ramesh R, Sant DG, Jorwekar SU, Jadhav MR, Tupe SG, et al. Silicon Incorporated Morpholine Antifungals: Design, Synthesis, and Biological Evaluation. ACS Med Chem Lett. 2015;6(11):1111–6. Epub 20150922. doi: 10.1021/acsmedchemlett.5b00245 . 26617963 PMC4645241

[43] Cirigliano A, Macone A, Bianchi MM, Oliaro-Bosso S, Balliano G, Negri R, et al. Ergosterol reduction impairs mitochondrial DNA maintenance in S. cerevisiae. Biochim Biophys Acta Mol Cell Biol Lipids. 2019;1864(3):290–303. Epub 20181212. doi: 10.1016/j.bbalip.2018.12.002 . 30553056

[44] Liu Q, Jiang S, Zheng K, Song J, Liang P. Interaction Between Amorolfine and Voriconazole Against Fusarium species. Mycopathologia. 2021;186(4):535–42. Epub 20210605. doi: 10.1007/s11046-021-00568-8 . 34089428

[45] Oliver JD, Sibley GEM, Beckmann N, Dobb KS, Slater MJ, McEntee L, et al. F901318 represents a novel class of antifungal drug that inhibits dihydroorotate dehydrogenase. Proc Natl Acad Sci U S A. 2016;113(45):12809–14. Epub 20161025. doi: 10.1073/pnas.1608304113 . 27791100 PMC5111691

[46] Jørgensen KM, Astvad KMT, Hare RK, Arendrup MC. EUCAST Determination of Olorofim (F901318) Susceptibility of Mold Species, Method Validation, and MICs. Antimicrob Agents Chemother. 2018;62(8). Epub 20180727. doi: 10.1128/AAC.00487-18 . 29784842 PMC6105844

[47] Wiederhold NP, Najvar LK, Jaramillo R, Olivo M, Birch M, Law D, et al. The Orotomide Olorofim Is Efficacious in an Experimental Model of Central Nervous System Coccidioidomycosis. Antimicrob Agents Chemother. 2018;62(9). Epub 20180827. doi: 10.1128/aac.00999-18 . 29941638 PMC6125497

[48] Rivero-Menendez O, Cuenca-Estrella M, Alastruey-Izquierdo A. In vitro activity of olorofim against clinical isolates of Scedosporium species and Lomentospora prolificans using EUCAST and CLSI methodologies. J Antimicrob Chemother. 2020;75(12):3582–5. doi: 10.1093/jac/dkaa351 . 32856079

[49] Garavito MF, Narváez-Ortiz HY, Zimmermann BH. Pyrimidine Metabolism: Dynamic and Versatile Pathways in Pathogens and Cellular Development. J Genet Genomics. 2015;42(5):195–205. Epub 20150508. doi: 10.1016/j.jgg.2015.04.004 . 26059768

[50] Gow NAR, Latge JP, Munro CA. The Fungal Cell Wall: Structure, Biosynthesis, and Function. Microbiol Spectr. 2017;5(3). doi: 10.1128/microbiolspec.FUNK-0035-2016 . 28513415 PMC11687499

[51] du Pré S, Birch M, Law D, Beckmann N, Sibley GEM, Bromley MJ, et al. The Dynamic Influence of Olorofim (F901318) on the Cell Morphology and Organization of Living Cells of Aspergillus fumigatus. J Fungi (Basel). 2020;6(2). Epub 20200410. doi: 10.3390/jof6020047 . 32290206 PMC7345704

[52] van Rhijn N, Hemmings S, Valero C, Amich J, Bromley MJ. Olorofim and the azoles are antagonistic in A. fumigatus and functional genomic screens reveal mechanisms of cross resistance. bioRxiv. 2021:2021.11.18.469075.

[53] Seyedmousavi S, Chang YC, Law D, Birch M, Rex JH, Kwon-Chung KJ. Efficacy of Olorofim (F901318) against Aspergillus fumigatus, A. nidulans, and A. tanneri in Murine Models of Profound Neutropenia and Chronic Granulomatous Disease. Antimicrob Agents Chemother. 2019;63(6). Epub 20190524. doi: 10.1128/AAC.00129-19 . 30885903 PMC6535528

[54] Seyedmousavi S, Chang YC, Youn JH, Law D, Birch M, Rex JH, et al. In Vivo Efficacy of Olorofim against Systemic Scedosporiosis and Lomentosporiosis. Antimicrob Agents Chemother. 2021;65(10):e0043421. Epub 20210712. doi: 10.1128/AAC.00434-21 . 34252298 PMC8448127

[55] Wiederhold NP. Review of the Novel Investigational Antifungal Olorofim. J Fungi (Basel). 2020;6(3). Epub 20200730. doi: 10.3390/jof6030122 . 32751765 PMC7557671

